# Development of a two-component recombinant vaccine for COVID-19

**DOI:** 10.3389/fimmu.2024.1514226

**Published:** 2024-12-20

**Authors:** Yi-Sheng Sun, Fang Xu, Han-Ping Zhu, Yong Xia, Qiao-Min Li, Yuan-Yuan Luo, Hang-Jing Lu, Bei-Bei Wu, Zhen Wang, Ping-Ping Yao, Zhan Zhou

**Affiliations:** ^1^ Zhejiang Key Lab of Vaccine, Infectious Disease Prevention and Control, Zhejiang Provincial Center for Disease Control and Prevention, Hangzhou, China; ^2^ Innovation Institute for Artificial Intelligence in Medicine and Zhejiang Provincial Key Laboratory of Anti-Cancer Drug Research, College of Pharmaceutical Sciences, Zhejiang University, Hangzhou, China; ^3^ The Fourth Affiliated Hospital, Zhejiang University School of Medicine, Yiwu, China

**Keywords:** COVID-19, vaccine, receptor-binding domain (RBD), N-terminal domain (NTD), Fc fusion

## Abstract

**Introduction:**

Though COVID-19 as a public health emergency of international concern (PHEIC) was declared to be ended by the WHO, it continues to pose a significant threat to human society. Vaccination remains one of the most effective methods for preventing COVID-19. While most of the antigenic regions are found in the receptor binding domain (RBD), the N-terminal domain (NTD) of the S protein is another crucial region for inducing neutralizing antibodies (nAbs) against COVID-19.

**Methods:**

In the two-dose immunization experiment, female BALB/c mice were intramuscularly immunized with different ratios of RBD-Fc and NTD-Fc proteins, with a total protein dose of 8 μg per mouse. Mice were immunized on day 0 and boosted on day 7. In the sequential immunization experiment, groups of female BALB/c mice were immunized with two doses of the inactivated SARS-CoV-2 vaccine (prototype strain) on day 0 and 7. On day 28, mice were boosted with RBD-Fc, NTD-Fc, RBD-Fc/NTD-Fc (9:1), RBD-Fc/NTD-Fc (3:1), inactivated SARS-CoV-2 vaccine (protoype strain), inactivated SARS-CoV-2 vaccine (omicron strain), individually. The IgG antibodies were detected using ELISA, while the neutralizing antibodies were measured through a microneutralization assay utilizing both the prototype and omicron strains. The ELISPOT assays were performed to measure the secretion of IL-4 and IFN-γ, and the concentrations of secreted IL-2 and IL-10 in the supernatants were measured by ELISA.

**Results:**

We have first developed a two-component recombinant vaccine for COVID-19 based on RBD-Fc and NTD-Fc proteins, with an optimal RBD-Fc/NTD-Fc ratio of 3:1. This novel two-component vaccine demonstrated the ability to induce durable and potent IgG antibodies, as well as the neutralizing antibodies in both the two-dose homologous and sequential vaccinations. Heterologous booster with this two-component vaccine could induce higher neutralizing antibody titers than the homologous group. Additionally, the vaccine elicited relatively balanced Th1- and Th2-cell immune responses.

**Conclusion:**

This novel two-component recombinant vaccine exhibits high immunogenicity and offers a potential booster strategy for COVID-19 vaccine development.

## Introduction

The novel coronavirus pneumonia (COVID-19), caused by the severe acute respiratory syndrome coronavirus 2 (SARS-CoV-2), has been ravaging the world over the past 3 years. Although COVID-19 as a public health emergency of international concern (PHEIC) was declared to have ended by the WHO on 5 May 2023 (1), the pandemic itself is still far from over. As an RNA virus, SARS-CoV-2 mutates easily, leading to the emergence of numerous variants capable of evading the humoral immune system (2, 3). In less than 4 years, the dominant strains have shifted from Alpha, Delta, to Omicron (4, 5). The pace of vaccine development could not keep up with the speed of virus mutation. COVID-19 remains a significant threat to human society and more COVID-19 vaccine candidates should be developed.

Recombinant subunit vaccines, with their advantage of large-scale production and transportation, have been widely used in the development of COVID-19 vaccines (6). Several COVID-19 subunit vaccines, such as ZF2001, are based on the receptor-binding domain (RBD) (7). While most of the antigenic regions are found in the RBD, the N-terminal domain (NTD) of the S protein is another crucial region for inducing neutralizing antibodies (nAbs) against COVID-19 (8, 9). Several human nAbs that bind to the NTD, such as COV2-2676 and COV2-2489, had been developed and demonstrated both effective prophylactic and therapeutic efficacy against SARS-CoV-2 infection (9–11). Moreover, macaques immunized with a combination of NTD and RBD immunogens could be totally protected from lethal SARS-CoV-2 challenges (12), highlighting the promising potential of the NTD in vaccine development.

Fc fusion proteins, which are composed of an immunoglobin Fc region and a desired linked protein, serve as an effective backbone for drug development (13). To date, 11 Fc fusion proteins have been approved by the Food and Drug Administration (FDA) (14). The Fc region could bind to the neonatal Fc receptor to prolong the plasma half-life and increase the immunogenicity of the Fc-fusion proteins (15). In our previous study, we developed RBD-Fc and RBD_delta_-Fc fusion vaccine candidates, both of which were able to induce humoral and cellular immune responses in mice (6, 16). This study aims to develop a two-component recombinant vaccine using RBD-Fc and NTD-Fc, optimizing their ratio to achieve enhanced humoral and cellular immune responses. It is the first combination of RBD-Fc and NTD-Fc for COVID-19 vaccine development. This vaccine was also applied in a sequential immunization after two doses of inactivated vaccination. Humoral responses such as immunoglobulin G (IgG) and nAb were monitored for 27 weeks. The IFN-γ- and IL-4-producing cells, as well as the secretion of IL-10 and IL-2, were also measured. Through these experiments, we demonstrated that the two-component vaccine based on the RBD-Fc and NTD-Fc proteins exhibited strong immunogenicity and might represent a promising candidate for the COVID-19 vaccine development.

## Materials and methods

### Cells and viruses

Vero E6 cells (NCACC) were cultured at 37℃ under 5% CO_2_ in Minimal Essential Medium (Gibco, Waltham, MA, USA) supplemented with 100 U/mL penicillin, 100 μg/mL streptomycin, and 10% fetal bovine serum (FBS). The prototype SARS-CoV-2 strain (12#) and the SARS-CoV-2 Omicron strain (BA.2) were obtained as mentioned previously (17, 18). Viruses were propagated in Vero E6 cells, and the 50% tissue culture infective dose (TCID_50_) was calculated by the Reed and Muench method (17).

### Mouse experiments

**Figure 1:**
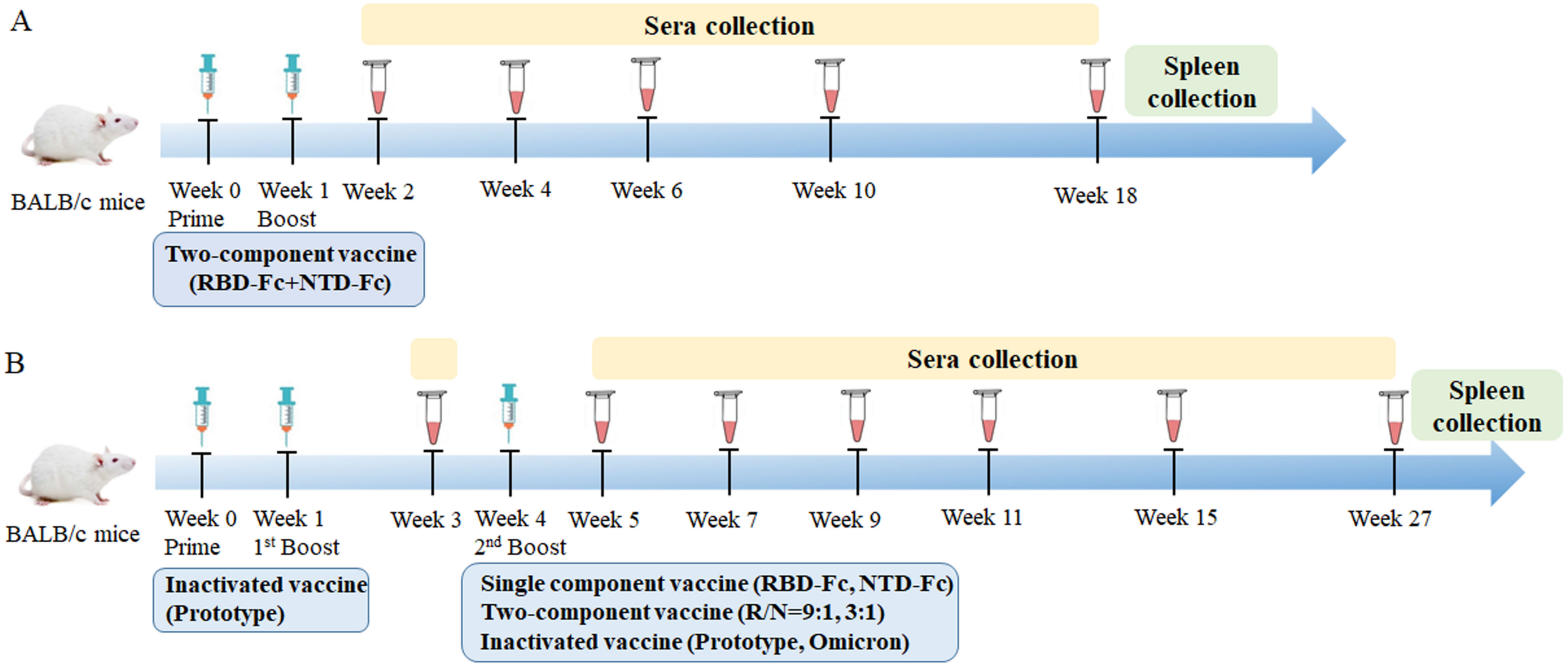
Schematic diagram of immunization and sera collection. **(A)** Two-dose immunization: BALB/c mice were immunized with different ratios (9:1, 3:1, 1:1, 8:0, 0:8, 1:3, and 1:9) of RBD-Fc and NTD-Fc proteins at week 0 and boosted at week 1. The sera were collected at weeks 2, 4, 6, 10, and 18. Spleens were harvested after euthanasia. **(B)** Sequential immunization: BALB/c mice were immunized with two doses of inactivated SARS-CoV-2 vaccine (prototype) at weeks 0 and 1. At week 4, mice were boosted with either RBD-Fc (8 μg), NTD-Fc (8 μg), RBD-Fc/NTD-Fc (9:1, 8 μg), RBD-Fc/NTD-Fc (3:1, 8 μg), inactivated SARS-CoV-2 vaccine (prototype), inactivated SARS-CoV-2 vaccine (omicron strain), or PBS. The sera were collected at weeks 3, 5, 7, 9, 11, 15, and 27. Spleens were collected after euthanasia.

In the two-dose immunization experiment, female BALB/c mice (8 weeks old, *n* = 5/group) were intramuscularly immunized with different ratios of RBD-Fc and NTD-Fc proteins (9:1, 3:1, 1:1, 8:0, 0:8, 1:3, and 1:9), with a total protein dose of 8 μg per mouse. Aluminum hydroxide was used as an adjuvant, mixed with immunized protein at a final solution of 0.5 mg/mL. Mice were immunized on day 0 and boosted on day 7, and phosphate buffer saline (PBS) containing 0.5 mg/mL of aluminum hydroxide was used as a negative control. The sera were collected as per the schedule shown in **Figure 1A**.

For the sequential immunization experiment, seven groups of female BALB/c mice (8 weeks old, *n* = 5/group) were immunized with two doses of the inactivated SARS-CoV-2 vaccine (prototype strain) on days 0 and 7. On day 28, mice were boosted with RBD-Fc (8 μg), NTD-Fc (8 μg), RBD-Fc/NTD-Fc (9:1, 8 μg), RBD-Fc/NTD-Fc (3:1, 8 μg), inactivated SARS-CoV-2 vaccine (prototype strain), inactivated SARS-CoV-2 vaccine (omicron strain), and PBS, individually. Mice receiving three doses of PBS were used as negative control. The sera and spleens were collected as per the schedule shown in **Figure 1B**. The RBD-Fc protein and the inactivated SARS-CoV-2 vaccine were prepared as previously described (6, 19). NTD-Fc was purchased from Sino Biological Company.

### Enzyme-linked immunosorbent assay

Briefly, enzyme-linked immunosorbent assay (ELISA) plates were coated with 50 ng of RBD or NTD or S1 protein per well overnight in carbonate-bicarbonate buffer (pH 9.6) and then blocked with 10% FBS in PBS. Serum samples were serially diluted twofold and added to each well. After three washes, the plates were incubated with rabbit anti-mouse IgG-HRP antibody at a dilution of 1:20,000 (Abcam, Cambridge, UK) for 1 h at 37℃. The color development reaction was performed using tetramethylbenzidine (TMB) and stopped with 2 M H_2_SO_4_. Absorbance was measured at 450 nm, and the endpoint dilution titer was defined as the highest reciprocal dilution of serum that produced an absorbance value at least 2.1 times above the background.

### Neutralization assay

Mouse serum was inactivated at 56℃ for 30 min. Serial 2-fold dilutions of the sera were incubated with 100 TCID_50_ of SARS-CoV-2 virus (prototype or omicron strain) for 1 h. The virus–serum mixtures were added to the Vero E6 cell-seeded 96-well plates and incubated for 3 days. Cytopathic effect (CPE) was observed in each well, and the neutralization titers were calculated as the reciprocal of the highest serum dilution than can protect 50% of wells from infection (16).

### Enzyme-linked immunospot assay

The enzyme-linked immunospot assay (ELISPOT) assays were performed according to the instructions of IL-4 and IFN-γ ELISPOT kits. Twenty-seven weeks after vaccination, mice in the sequential immunization groups were euthanized, and their spleens were collected. Splenocytes were isolated by pressing spleens through the cell strainers and cultured in ELISPOT plates at a density of 2 × 10^5^ per well for the IFN-γ and IL-4 detection. A cocktail containing RBD (1 μg/well) and NTD (1 μg/well) was used as the stimulant. Spot-forming cells (SFCs) were imaged with a ChemiDoc XRS+ imaging system (Bio-Rad, CA, USA) and analyzed using the Quantity One software.

### IL-2 and IL-10 detection

Splenocytes were prepared as described in the ELISPOT section and seeded in a 96-well plate at a density of 2 × 10^5^ per well. After stimulation with RBD-NTD mixture (1:1, 2 μg/well) for 16 h, the supernatants of each well were obtained and the concentrations of secreted IL-2 and IL-10 were measured by ELISA kits (BD Biosciences, NJ, USA).

### Statistical analysis

All data were analyzed with GraphPad Prism 8.0. The Student’s *t*-test was used for comparisons between two groups, and *p* < 0.05 was considered as statistically significant.

## Results

### The immunogenicity of the two-component vaccines

**Figure 2:**
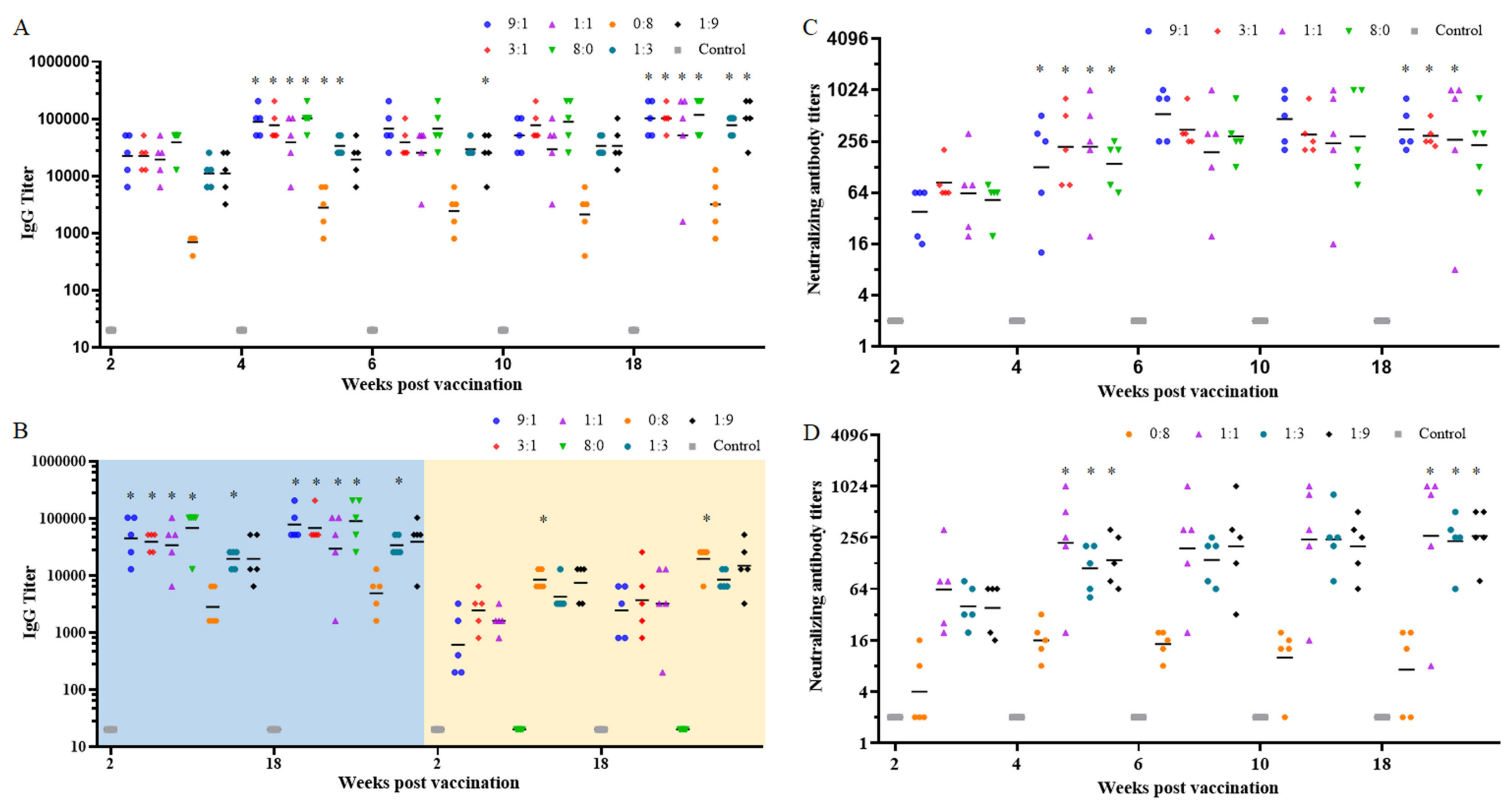
The immunogenicity of the two-component vaccines. BALB/c mice were immunized with various ratios of RBD-Fc and NTD-Fc proteins (9:1, 3:1, 1:1, 8:0, 0:8, 1:3, and 1:9) at week 0 and boosted at week 1. Sera collected at weeks 2, 4, 6, 10, and 18 were analyzed by ELISA using plates coated with RBD **(A)** and NTD or S1 **(B)**. The neutralizing antibody titers in sera from the RBD-Fc/NTD-Fc (9:1, 3:1, 1:1, and 8:0) groups **(C)** and the RBD-Fc/NTD-Fc (0:8, 1:1, 1:3, and 1:9) groups **(D)** were measured using authentic virus (prototype). PBS containing 0.5 mg/mL of aluminum hydroxide was used as a negative control. *p < 0.05.

To determine the optimal RBD-Fc/NTD-Fc ratio for immunization, different RBD-Fc/NTD-Fc (R/N) ratios (9:1, 3:1, 1:1, 8:0, 0:8, 1:3, and 1:9) with a total protein amount of 8 μg for each mouse were tested. Mice were immunized and boosted after 1 week. Two weeks following the initial immunization, the IgG seroconversion rate was 100% in all groups (**Figure 2A**). However, the geometric mean titer (GMT) of IgG antibodies in the R/N (0:8) group was 696, significantly lower than the other groups. Four weeks post-vaccination, IgG levels in all groups increased sharply. The GMTs of IgG antibodies in the R/N (9:1, 3:1, 1:1, 8:0, 0:8, and 1:3) groups were 89,144, 77,604, 38,802, 102,400, 2,786, and 33,779, significantly higher than those of each corresponding group at 2 weeks post-vaccination. In the R/N (1:9) group, the GMT of IgG antibodies was 19,401 at 4 weeks post-vaccination, rising to 25,600 2 weeks later, which was significantly higher than that at 2 weeks post-vaccination. The high IgG levels could last for at least 18 weeks. The GMTs of IgG antibodies in the R/N (9:1, 3:1, 1:1, 8:0, 0:8, 1:3, and 1:9) groups at 18 weeks post-vaccination were 102,400, 102,400, 51,200, 117,627, 3,200, 77,605 and 102,400, respectively.

In addition to the RBD protein, S1 and NTD proteins were also coated onto the microwells of ELISA plates. As shown in **Figure 2B**, the GMTs of IgG antibodies in the R/N (9:1, 3:1, 1:1, 8:0, and 1:3) groups using S1-coated plates (left, blue background) were significantly higher than those using the NTD-coated plates (right, yellow background) at 2 weeks post-vaccination. Furthermore, the GMT of IgG antibodies in the R/N (0:8) groups using S1-coated plates was significantly lower than that using the NTD-coated plates at 2 weeks post-vaccination. Similar results were observed at 18 weeks post-vaccination. Using S1-coated plates, the GMTs of IgG antibodies in the R/N (9:1, 3:1, 1:1, 8:0, 0:8, 1:3, and 1:9) groups at 18 weeks post-vaccination were 77,605, 67,559, 29,407, 89,144, 4,850, 33,779, and 38,802, respectively. Among all groups, the GMTs of IgG antibodies in the R/N (9:1, 3:1, and 8:0) groups were relatively high when using both RBD- and S1-coated plates.

To further evaluate the immunogenicity of the two-component vaccines, we measured the titers of nAbs using the prototype SARS-CoV-2 virus. The seroconversion rates of nAbs in the R/N (9:1, 3:1, 1:1, 8:0, 1:3, and 1:9) groups were 100% at 2 weeks post-vaccination, while that in the R/N (0:8) group was only 40% (**Figures 2C, D**). Similar to the IgG titers, the nAb titers in all groups increased sharply at 4 weeks post-vaccination. By this time, the seroconversion rate of nAbs in the R/N (0:8) group had risen to 100%, and the GMTs of nAbs in R/N (9:1, 3:1, 1:1, 8:0, 1:3, and 1:9) groups were significantly higher than those of the corresponding groups at 2 weeks post-vaccination. The GMTs of nAbs in R/N (9:1, 3:1, 1:1, 8:0, 0:8, 1:3, and 1:9) groups were 127, 221, 222, 140, 16, 112, and 139, respectively, with the R/N (3:1, 1:1) groups showing the highest levels. At 18 weeks post-vaccination, the GMTs of nAbs in the R/N (9:1, 3:1, 1:1, 8:0, 0:8, 1:3, and 1:9) groups were 354, 299, 269, 231, 17, 232, and 267, respectively, with the R/N (9:1, 3:1) groups exhibiting slightly higher titers compared to the others. Considering the GMTs of IgG antibodies and nAbs at 18 weeks post-vaccination, we selected the RBD-Fc/NTD-Fc ratios of 9:1 and 3:1 for the two-component vaccines.

### The humoral immune response in the sequential immunization

**Figure 3:**
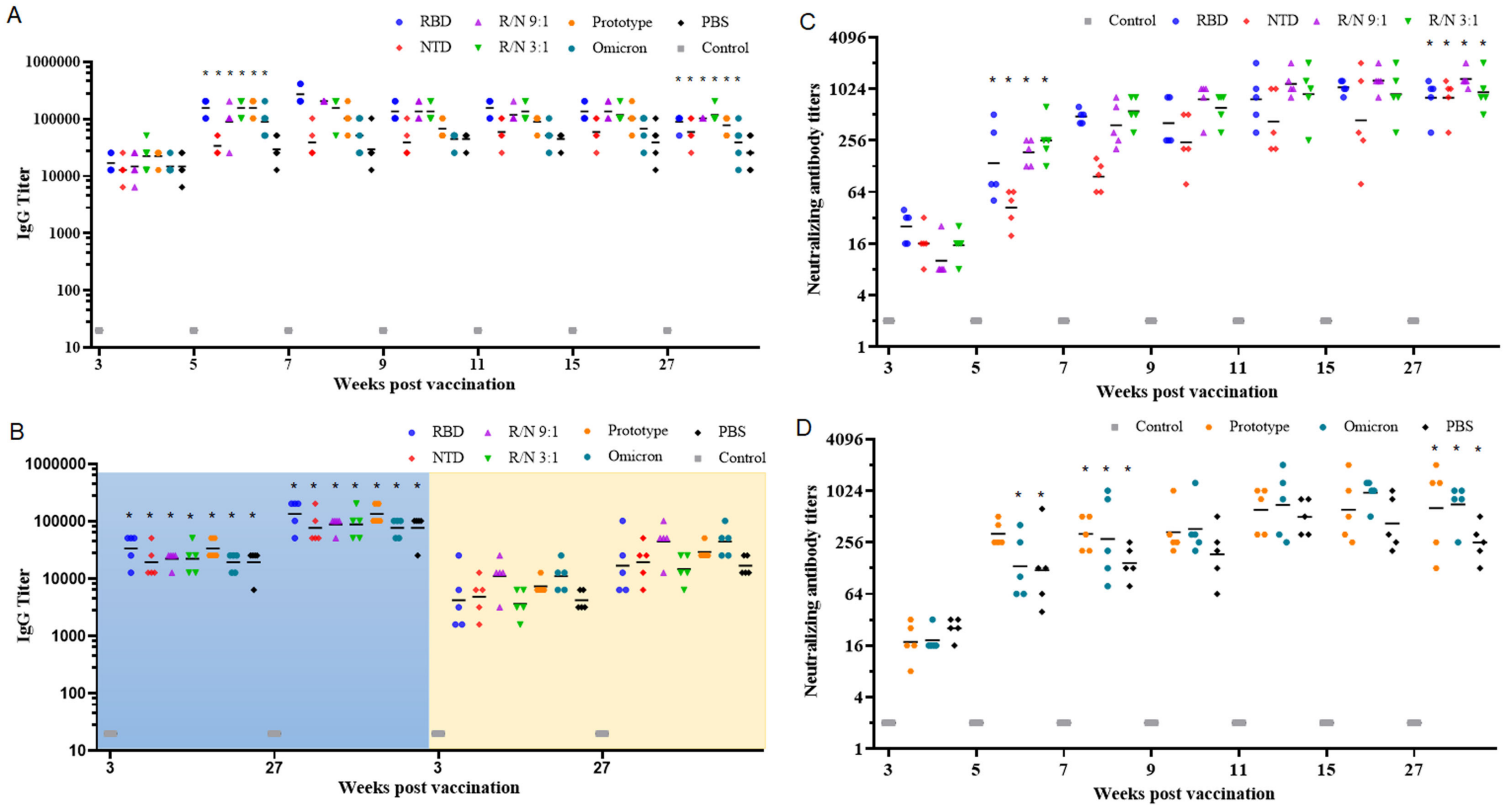
Sequential immunization with the two-component vaccines effectively improve the humoral immune response. BALB/c mice were immunized with two doses of inactivated SARS-CoV-2 vaccine (prototype) at weeks 0 and 1. At week 4, mice were boosted with either RBD-Fc, NTD-Fc, RBD-Fc/NTD-Fc (9:1), RBD-Fc/NTD-Fc (3:1), inactivated SARS-CoV-2 vaccine (prototype), inactivated SARS-CoV-2 vaccine (omicron strain), or PBS. Sera collected at weeks 3, 5, 7, 9, 11, 15, and 27 were analyzed by ELISA using RBD-coated plates **(A)**, and NTD or S1-coated plates **(B)**. The neutralizing antibody titers in the RBD, NTD, R/N (9:1), and R/N (3:1) groups **(C)**, as well as the prototype, omicron, and PBS groups **(D)**, were measured using live prototype virus. Mice immunized with three doses of PBS containing 0.5 mg/mL of aluminum hydroxide were used as the negative control. *p < 0.05.

To verify the effectiveness of the two-component vaccines in the heterologous immunization, mice were first immunized with two doses of prototypic inactivated SARS-CoV-2 vaccines on day 0 and day 7. Subsequently, the mice received a booster with RBD-Fc (8 μg), NTD-Fc (8 μg), R/N (9:1, 8 μg), R/N (3:1, 8 μg), the prototypic inactivated SARS-CoV-2 vaccine, the inactivated omicron vaccine, or PBS on day 28. Five weeks post-vaccination (1 week after the third immunization), the GMTs of IgG antibodies in the RBD, NTD, R/N (9:1, 3:1), prototype, and omicron groups were all significantly higher than those at 3 weeks post-vaccination (1 week after the second immunization). The elevated levels of IgG antibodies persisted for at least 22 weeks, with GMTs of 89,144, 51,200, 102,400, 117,626, 77,605, and 77,605 for the RBD, NTD, R/N (9:1), R/N (3:1), prototype, and omicron groups, respectively (**Figure 3A**). Using S1-coated ELISA plates (left, blue background), the GMTs of IgG antibodies in the RBD, NTD, R/N (9:1, 3:1), prototype, and omicron groups were also high, similar to the results obtained using RBD-coated plates (**Figure 3B**). However, compared with the results from NTD-coated plates (right, yellow background), the GMTs of IgG antibodies in all groups using S1-coated plates were higher at both 3 and 27 weeks post-vaccination.

Similar to the IgG antibody titers, the nAbs against the prototype SARS-CoV-2 virus were also increased significantly at 1 week after the third vaccination. The GMTs of nAbs in the RBD, NTD, R/N (9:1, 3:1), prototype, and omicron groups were 139, 42, 185, 255, 323, and 134, respectively. The GMTs of nAbs continued to rise over time, peaking at 15 weeks post-vaccination. These elevated nAb titers could sustain for at least 22 weeks. At 27 weeks post-vaccination, the GMTs of nAbs in the RBD, NTD, R/N (9:1), R/N (3:1), prototype, omicron, and PBS groups were 806, 806, 1,333, 935, 639, 708, and 255, respectively, still significantly higher than those at 3 weeks post-vaccination. Based on the results of IgG and nAbs, both the RBD-Fc/NTD-Fc ratios of 9:1 and 3:1 appeared to be suitable for the two-component vaccine.

### The neutralizing antibody against the SARS-CoV-2 omicron variant

**Figure 4:**
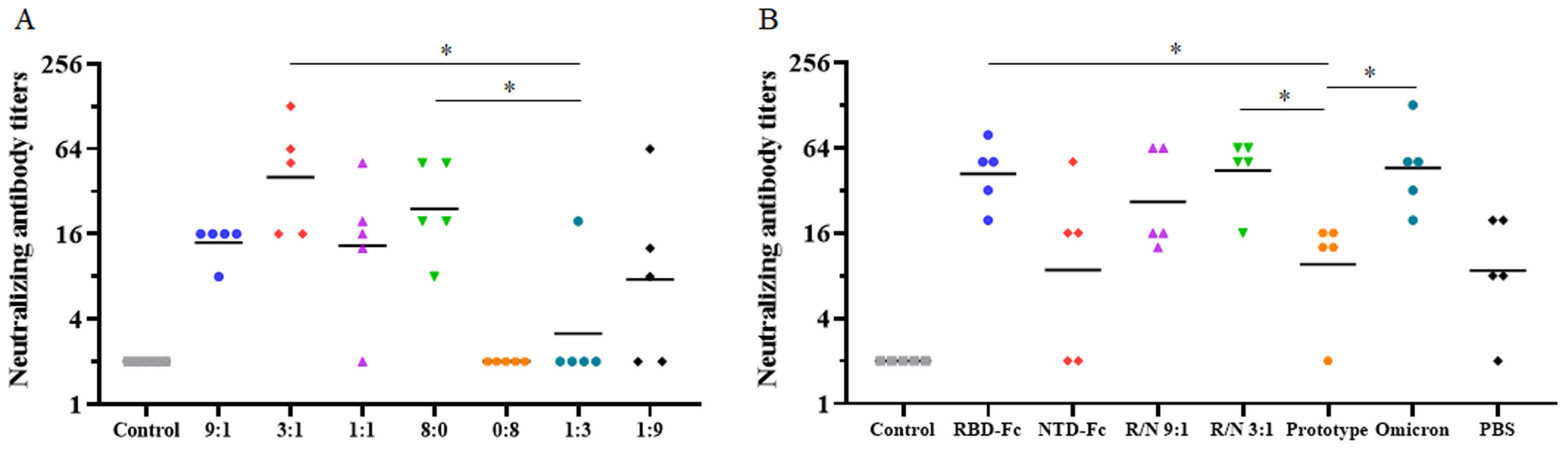
The neutralizing antibody against the SARS-CoV-2 Omicron variant. **(A)** The neutralizing antibody titers of the R/N (9:1, 3:1, 1:1, 8:0, 0:8, 1:3, and 1:9) groups in the two-dose immunization experiment were measured using the Omicron variant (BA.2). PBS containing 0.5 mg/mL of aluminum hydroxide was used as a negative control. **(B)** Neutralizing antibody titers of the RBD, NTD, R/N (9:1), R/N (3:1), prototype, omicron, and PBS groups in the sequential immunization experiment were measured by the Omicron variant (BA.2). Mice immunized with three doses of PBS were used as the negative control. *p < 0.05.

The omicron variant (BA.2) was also utilized to assess the nAb levels in both the two-dose immunization and the sequential immunization. In the two-dose immunization, the GMTs of nAbs at 18 weeks post-vaccination in the R/N (9:1, 3:1, 1:1, 8:0, 1:3, and 1:9) groups were 14, 40, 13, 24, 3, and 8 (**Figure 4A**). Notably, nAbs were undetectable in the R/N (0:8) group. The GMTs of nAbs in R/N (3:1 and 8:0) groups were both significantly higher than those in the R/N (1:3) group. In the sequential immunization, the GMTs of nAbs at 27 weeks post-vaccination in the RBD, NTD, R/N (9:1), R/N (3:1), prototype, omicron, and PBS groups were 42, 9, 27, 44, 10, 46, and 9, respectively (**Figure 4B**). The GMTs of nAbs in RBD, R/N (3:1), and omicron groups were significantly higher than those in R/N (1:3) groups. Overall, these results suggested that the R/N (3:1) group might induce a relatively high level of nAbs compared to the other groups, making it a potential candidate for the two-component vaccine.

### The cellular immune response in the sequential immunization

**Figure 5:**
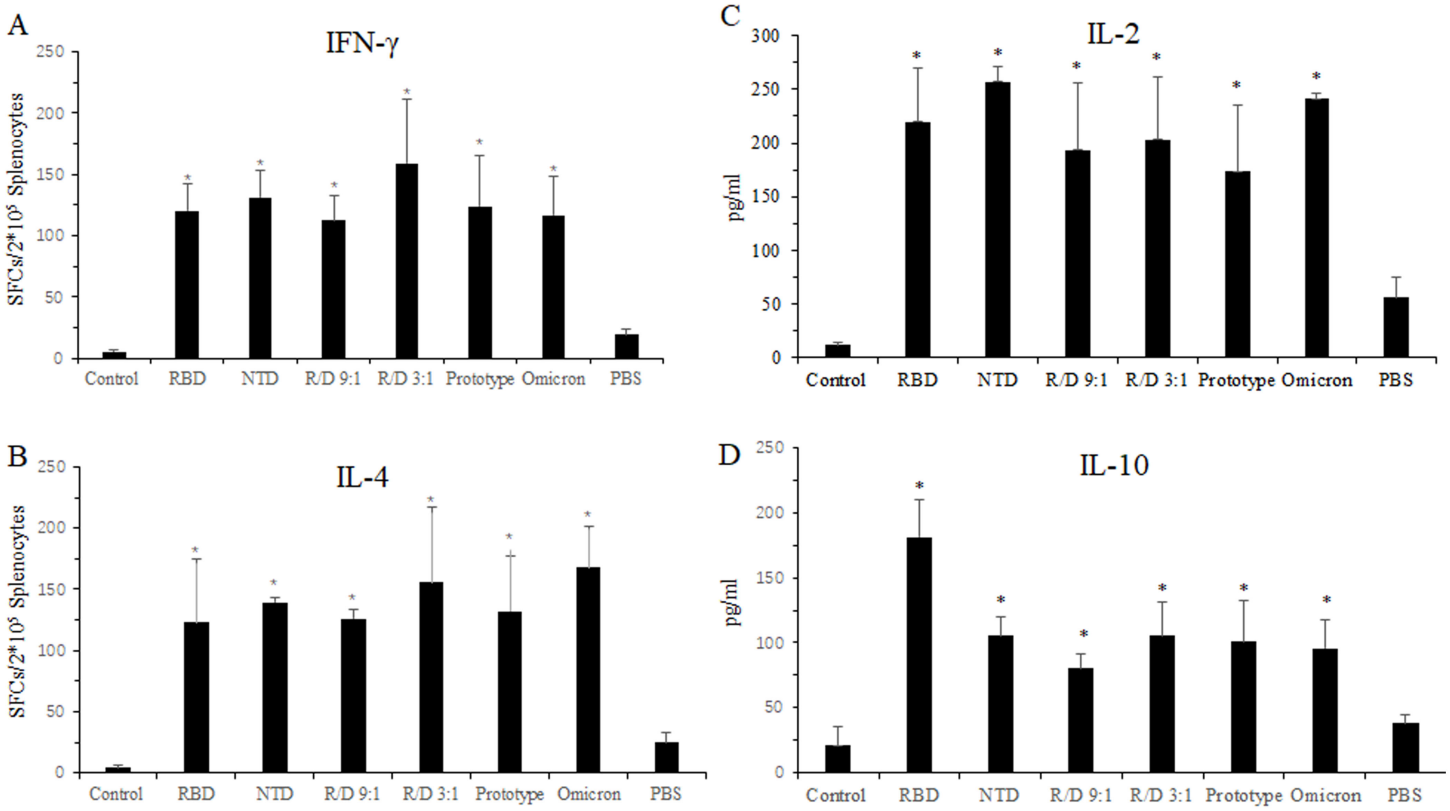
Cellular immune response in the sequential immunization. Twenty-seven weeks after vaccination, the splenocytes from the mice in RBD-Fc, NTD-Fc, RBD-Fc/NTD-Fc(9:1), RBD-Fc/NTD-Fc(3:1), inactivated SARS-CoV-2 vaccine (prototype), inactivated SARS-CoV-2 vaccine (omicron strain), and PBS groups were stimulated with a cocktail of the RBD and NTD proteins for 16 h Spleens were collected at week 27 after euthanasia. The IFN-γ-producing cells **(A)** and the IL-4-producing cells **(B)** were quantified using IFN-γ and IL-4 ELISPOT assays, respectively. The concentrations of IL-2 **(C)** and IL-10 **(D)** in the splenocyte culture medium were detected by ELISA. Mice immunized with three doses of PBS were used as the negative control. *p < 0.05.

Next, we evaluated the cellular immune response of the two-component vaccines in the sequential immunization using ELISPOT assay. Compared to the PBS group, a significantly higher number of IFN-γ SFCs in the RBD, NTD, R/N (9:1), R/N (3:1), prototype, and omicron groups were found, with counts of 120, 131, 113, 159, 124, and 117, respectively (**Figure 5A**). Similar results were found from the IL-4 ELISPOT assay, where the number of IL-4 SFCs was greater in the RBD, NTD, R/N (9:1), R/N (3:1), prototype, and omicron groups compared to the PBS group (**Figure 5B**).

We also measured the levels of IL-2 and IL-10 secreted by the splenocytes of immunized mice using ELISA. As shown in **Figures 5C, D**, the concentrations of IL-2 and IL-10 in the three dose groups such as RBD, NTD, R/N (9:1), R/N (3:1), prototype, and omicron groups were higher than those in the two dose PBS group. The IL-10 concentrations of the RBD, NTD, R/N (9:1), R/N (3:1), prototype, and omicron groups were 181.2, 105.3, 80.3, 105.3, 100.8, and 95.8, respectively. The IL-2 concentrations of those groups were 219.7, 257.3, 193.1, 203.2, 173.5, and 241.1.

All results showed that a third vaccination could effectively stimulate a robust cellular immune response.

## Discussion

While COVID-19 is no longer classified as a public health emergency, it is still a significant challenge to the whole society. Here, we have first developed a two-component recombinant vaccine for COVID-19 based on RBD-Fc and NTD-Fc proteins, with an optimal RBD-Fc/NTD-Fc ratio of 3:1. This two-component vaccine is capable of inducing durable and potent IgG and nAbs in both the homologous and heterologous immunization experiments. Additionally, it effectively triggers robust cellular immune response. Overall, this two-component vaccine exhibits great potential as a COVID-19 vaccine candidate.

The design of immunogens is critical for the vaccine development. Both RBD and NTD were located at the S1 subunit of spike (S) protein, which binds to the host cell receptor, angiotensin-converting enzyme 2 (ACE2), facilitating viral entry into cell (20). The RBD, as the receptor binding site for the ACE2 receptor, is an ideal immunogen, and over 11 COVID-19 vaccines, including ZF2001 (Zhifei) and CoVaccine (WestVac), have been developed based on the RBD protein (21, 22). In contrast, the NTD has been less extensively investigated and used for vaccine development. Cumulative evidence indicates that lots of neutralizing epitopes are present in the NTD. nAbs such as 4A8, have been identified, along with specific epitope sites such as D144‐Q158 and E246‐T253 within the NTD (23, 24). Analysis of memory B cells from SARS-CoV-2-infected individuals had shown that anti-NTD antibodies could neutralize SARS-CoV-2 variants including Omicron (25). Additionally, the NTD plays a crucial role in host receptor recognition. Díaz-Salinas (26) reported that the NTD bound terminal sialic acid to enhance both SARS-CoV-2 S-mediated virus attachment and ACE2 binding. Mouse hepatitis coronavirus, a betacoronavirus, utilizes the NTD to recognize its host receptor, CEACAM1a (27). These findings suggested that the NTD might be another valuable immunogen alongside the RBD. Inclusion of NTD could enhance the immunogenicity of the RBD subunit vaccine, and fusing the NTD onto the RBD–RBD protein enhances T-cell immunity, which, in turn, supports B-cell immunity and overall improves the immunogenicity of the antigen (28). A cocktail of NTD-directed and RBD-targeting nAbs could also work synergistically to provide protection against SARS-CoV-2 (12). In the two-dose immunization assay, the GMTs of nAbs in the two-component vaccine groups (R/N = 9:1, 3:1, and 1:1) were 354, 299, and 269 at 18 weeks post-vaccination, slightly higher than those in the single-component vaccine group (RBD-Fc, R/N = 8:0; NTD-Fc, R/N = 0:8). Similar trends were also found in the heterologous immunization. The GMTs of nAbs in the two-component vaccine groups (R/N = 9:1, 3:1) were 1,333 and 935, compared to those of 806 and 805 in the RBD-Fc and NTD-Fc vaccine groups. When using the omicron strain in the neutralization assay, the GMT of nAbs in the R/N = 3:1 group was 40, which was still slightly higher than that of 24 in the RBD-Fc vaccine group. These results indicated that the two-component vaccine, consisting of RBD-Fc and NTD-Fc immunogens, exhibited robust immunogenicity than the single component vaccine at the same dose. The combination of NTD-Fc and RBD-Fc might have a synergistic effect on the immune system. Furthermore, when NTD-Fc was used as a single component vaccine, the GMT of nAbs in the two-dose immunization was only 17 at 18 weeks post-vaccination, significantly lower than those in the two-component vaccine groups and the RBD-Fc vaccine group. Although NTD contains several neutralizing epitopes, it may not be advisable to use NTD-Fc as the sole immunogen.

Since the S1 subunit comprises both the RBD and NTD, it is normal to question why we do not use S1 instead of RBD and NTD for vaccine development. However, in our previous study, we found that the S1-Fc protein exhibited poorer immunogenicity compared with the RBD-Fc group (6). Similar results were also found when comparing the S1 protein with the RBD protein (29). One reason for this might be that the two distinct proteins expose more key epitopes than a single combined protein, as some epitopes could be obscured by conformation changes. Although RBD and NTD were both located at the S1 subunit, S1 might not be an ideal immunogen for vaccine development.

As a two-component vaccine, the ratio of the RBD-Fc and NTD-Fc is a key parameter. The combined RBD and NTD immunogens with the ratio of 1:1 exhibited more robust immunogenicity than a single RBD or NTD immunogen at the same dose (12). Furthermore, the nAbs induced by RBD were generally more potent than those induced by NTD (25). The 1:1 ratio of RBD to NTD might not be optimal for immunization. Therefore, we tested additional ratios, including 9:1, 3:1, 1:3, and 1:9. In the two-dose immunization, the GMTs of IgG antibodies and nAbs in the R/N (9:1 and 3:1) groups were slightly higher than the R/N (1:1, 1:3, and 1:9) groups. In the heterologous immunization using the omicron variant for detection, the GMT of nAbs in the R/N (3:1) group was 44, compared to 27 in the R/N (9:1) group. Among all tested ratios, the RBD-Fc/NTD-Fc 3:1 group exhibited the highest sustained nAb titers, suggesting its optimal immunogenicity, while the RBD monomer has low immunogenicity, and the RBD dimer could enhance the immunogenicity (7, 30). By conjugating the human IgG Fc fragment to the C-terminus of the RBD protein, we previously constructed an RBD-Fc fusion protein, which exhibited superior immunogenicity in mice than the RBD protein. Fc might promote vaccine uptake by antigen-presenting cells (APCs), boosting the RBD- or NTD-stimulated immune capacity (16).

In this study, we first introduced NTD-Fc combined with RBD-Fc as a two-component vaccine. This combination was able to induce potent and durable nAbs against SARS-CoV-2, as well as a robust cellular immune response. Moreover, no significant damage was detected in the lung slices of the immunized mice (**Supplementary Figure S1**), indicating the safety of this two-component vaccine.

Though COVID-19 has become endemic, the SARS-CoV-2 virus continues to evolve to adapt the herd immunity (31). As of the summer of 2024, COVID-19 cases surged worldwide, with subvariants such as JN.1 and KP.3 displaying significant immune evasion (32). Nevertheless, vaccine is still one of the most effective measures to combat COVID-19. Given that most individuals in China have received at least two vaccinations, heterologous booster seems to be a promising vaccination strategy (33). In our study on heterologous immunization (**Figure 4B**), the two-component vaccine (R/N = 3:1) and the RBD-Fc vaccine induced significantly higher GMT of nAbs than that of the prototype inactivated vaccine (homologous group) when using the omicron strain. The GMTs of nAbs in the third immunization groups (RBD, NTD, R/N = 9:1, R/N = 3:1, prototype, and omicron) were significantly higher than those in the PBS group that received only two doses of the inactivated vaccine (**Figures 3C, D**). This indicated that an additional booster, particularly a heterologous booster, is beneficial.

Cellular immune response is pivotal for the vaccine efficacy, providing long-term protection for COVID-19 (34). In our study, results demonstrated that a second booster could trigger a significantly cellular immune response in both the homologous and heterologous immunization (**Figure 4**). The secretion of IFN-γ and IL-2 produced by Th1-cells, as well as IL-4 and IL-10 produced by Th-2 cells, was found to be increased. It seemed that the two-component vaccine induced a relatively balanced Th1- and Th2-cell immune responses.

In summary, our study is the first to employ the RBD-Fc and NTD-Fc as a two-component vaccine. This formulation demonstrated the ability to elicit high levels of IgG and nAbs, which could last for more than 6 months. The vaccine also induces a robust cellular immune response, and the optimal ratio of RBD-Fc to NTD-Fc was identified as 3:1. As the need for diverse COVID-19 vaccines remains critical in anticipation of future outbreaks, this two-component vaccine might be a promising candidate for further COVID-19 vaccine development.

## Data Availability

The original contributions presented in the study are included in the article/**Supplementary Material**. Further inquiries can be directed to the corresponding authors.
