# Supplementary material for: Development of a two-component recombinant vaccine for COVID-19
Source: Front Immunol. 2024 Dec 20;15:1514226. doi: 10.3389/fimmu.2024.1514226 (PMC11695372; doi:10.3389/fimmu.2024.1514226)
Supplement: Supplementary file 1 [file Table1.docx]

**Development of a two-component recombinant Vaccine for COVID-19**

**Yi-Sheng Sun^1^, Fang Xu^1^, Han-Ping Zhu^1^, Yong Xia^1^, Qiao-Min Li^2^, Yuan-Yuan Luo^2^, Hang-Jing Lu^1^, Bei-Bei Wu^1^, Zhen Wang^1*^, Ping-Ping Yao^1*^, Zhan Zhou^2,3*^**

^1^Zhejiang Key Lab of Vaccine, Infectious Disease Prevention and Control, Zhejiang Provincial Center for Disease Control and Prevention, Hangzhou 310015, China

^2^Innovation Institute for Artificial Intelligence in Medicine and Zhejiang Provincial Key Laboratory of An-ti-Cancer Drug Research, College of Pharmaceutical Sciences, Zhejiang University, Hangzhou 310058, China

^3^The Fourth Affiliated Hospital, Zhejiang University School of Medicine, Yiwu, 322000, China

**^*^Correspondence:** Zhen Wang, Email: wangzhen@cdc.zj.cn, Ping-Ping Yao, Email: ppyao@cdc.zj.cn, Zhan Zhou, Email: zhanzhou@zju.edu.cn.

**Supplementary Materials**


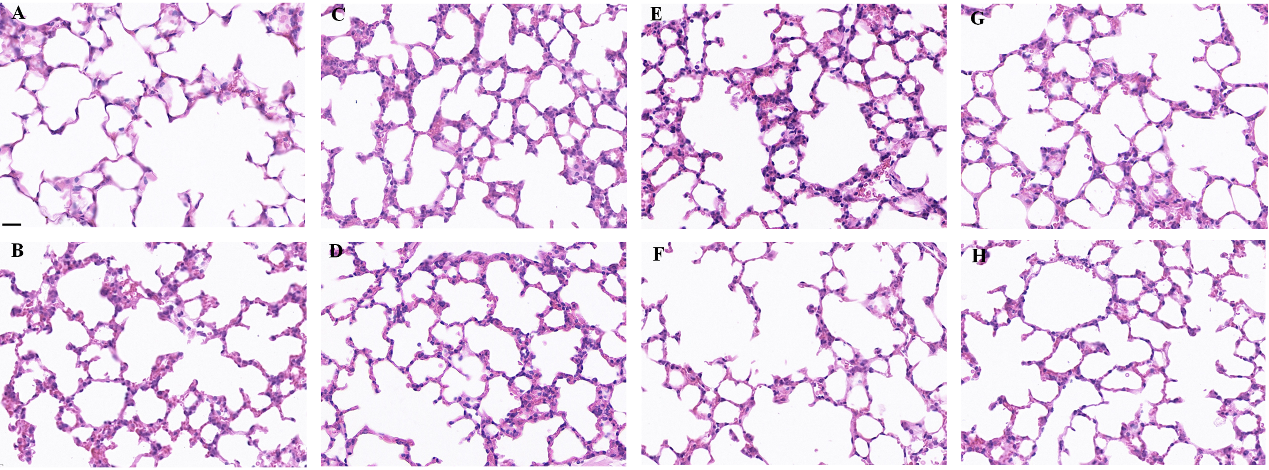


**Figure S1.** **Representative images of haematoxylin and eosin stained lungs isolated from the mice in the heterologous immunization.** BALB/c mice were immunized with two doses of inactivated SARS-CoV-2 vaccine (prototype,) at week 0 and 1. At week 4, mice were boosted with either RBD-Fc (A), NTD-Fc (B), RBD-Fc/NTD-Fc (9:1, C), RBD-Fc/NTD-Fc (3:1, D), inactivated SARS-CoV-2 vaccine (prototype, E), inactivated SARS-CoV-2 vaccine (omicron strain, F), or PBS (G). Mice were immunized with three dose of PBS were used as the negative control (H). Spleens were collected after euthanasia at 27 weeks post vaccination. Bar: 20 μm.
